# Direct and indirect short-term aggregated turbine- and farm-level wind power forecasts integrating several NWP sources

**DOI:** 10.1016/j.heliyon.2023.e21479

**Published:** 2023-10-27

**Authors:** Ghali Yakoub, Sathyajith Mathew, Joao Leal

**Affiliations:** University of Agder, Jon Lilletunsvei 9, 4879, Grimstad, Norway

**Keywords:** Wind power forecast (WPF), Aggregated turbine-level, Farm-level, NWP, Machine learning (ML)

## Abstract

The wind power sector is experiencing rapid growth, which creates new challenges for its electricity grid integration. Accurate wind power forecasting (WPF) is crucial for trading, balancing, and dispatching wind energy. In this paper, we examine the use of aggregated turbine- and farm-level WPFs in the Nordic energy market. The turbine-level WPFs were retrieved from a previous study, while the farm-level WPFs were developed using the same methodology, incorporating inputs from three different numerical weather predictions (NWPs) and implementing both direct and indirect forecasting approaches. In the indirect WPF approach, we explore the impact of using wind direction as an input for the wind farm-level power performance model. The different WPFs are combined into one using weights related to up-to-date forecast errors. An automated and optimized machine-learning pipeline using data from a Norwegian wind farm is used to implement the proposed forecasting methods. The indirect approach, that uses the wind-downscaling model, improves the wind speed forecast accuracy compared to raw forecasts from the relevant NWPs. Additionally, we observed that the farm-level downscaling model exhibited lower error than those developed at the turbine level. The combined use of multiple NWP sources reduced forecasting errors by 8 %–30 % for direct and indirect WPFs, respectively. Direct and indirect forecasting methods present similar performance. Finally, the aggregated turbine-level improved WPF accuracy by 10 % and 15 % for RMSE and MAE, respectively, compared to farm-level WPF.

## 1 Introduction

As the penetration of wind power into the electricity grid increases, accurate wind power forecasting (WPF) becomes critical for energy balancing, dispatching, and trading, particularly in regions where wind-generated electricity represents a sizable portion of the power supply. The temporal resolution of WPF varies with the specific application for which it is intended for. Long-term WPFs, ranging from days to weeks ahead, are important for wind farms’ maintenance planning. Similarly, short-to medium-term forecasts, targeting the generation from few minutes to hours ahead, are essential for efficient power dispatch and successful integration of wind energy in the electricity markets. The forecasting window for electricity markets was traditionally a day ahead, whereas, for example, in the NordPool markets, the daily forecasts have to be made 36 h before (i.e. at 12:00 UTC the day before). The prevalence of wind-generated electricity has caused a shift in the trading market towards shorter timeframes compared to the traditional day-ahead market. In the short-term market, participants modify their day-ahead bids based on intraday forecasts to mitigate the risk of incurring imbalance costs. In addition, system operators utilize these forecasts to ensure the security and reliability of the system by maintaining a real-time balance between supply and demand [1,2]. Energy providers may face additional project operating costs due to imbalance costs resulting from discrepancies between expected and actual wind power output. Accurate wind power forecasts can help mitigate these penalties, but eliminating them may not be possible [3] entirely. Apart from reducing the imbalance costs and penalties, reliable wind power forecasting can also help in efficient market integration of wind power, optimizing project development and operations, minimizing power curtailments and reducing the impact on the system under extreme weather conditions [3]. Though it will be challenging to generalize and quantify the financial benefits of these forecasts, some reported cases [4] indicate the potential of improved WPF to reduce costs. In that case, reducing the normalized mean absolute error (NMAE) by 1 % could reduce the annual generation costs in the Public Service of Colorado by over $1 million.

While high accuracy levels are desirable, it is important to note that all WPF models are susceptible to errors due to the wind's stochastic nature [5]. Accuracies of WPF, which are critical in choosing the best predictive approach, are to be assessed by several error metrics. Acceptable accuracy levels of the forecasting approaches reported in the literature vary depending upon their application. For example, it has been mentioned that the normalized root mean squared error (NRMSE) should be within 13 % of installed capacity for the first 6 h, which can go up to 22 % for 48 h ahead [6]. The State Grid Corporation in China has established a maximum acceptable NRMSE of 20 % for short-term wind power forecasting and a target accuracy of 15 % for the forecasted value of 4 h ahead. On the other hand, in Ireland a higher target accuracy range of 6–8% has been set [5]. To realize the targeted higher accuracies, the WPF field has evolved over the past decades, achieving higher accuracies, which are crucial for supporting energy balance and dispatching decisions in power generation [5,7].

WPF methods can be split in two main approaches: deterministic and probabilistic. Deterministic approaches offer a singular wind power prediction at a given time horizon. Recent research has shown that deterministic methods have improved significantly in terms of accuracy [5,7,8]. However, as wind power is stochastic in nature, probabilistic methods are also used for uncertainty analysis in future wind power generation. Probabilistic methods provide a confidence level that quantifies this uncertainty [[9], [10], [11]]. Being the most common approach [1,7,12], and as WPF users still prefer a single-valued estimate of the future power production due to the difficulties in interpreting probabilistic forecasts, deterministic forecasts are adopted in the present study. In general, deterministic forecasting models can be classified into four categories: physical [[13], [14], [15]], statistical [[16], [17], [18]], intelligent [[19], [20], [21], [22]] and hybrid [23,24]. Among these, the use of intelligent forecasting models have increased significantly in recent years, mainly because of machine learning and artificial intelligence advancements. These models are becoming increasingly popular due to their robustness and effectiveness, making them a promising option for handling nonstationary wind energy series [7].

WPF can be achieved through either direct or indirect frameworks. The traditional direct approach involves predicting wind power using time-series analysis without incorporating wind speed data [25,26]. While it has the advantage of not requiring the development of models that correlate the relationship between wind speed and wind power, its prediction accuracy may not always be sufficient due to the randomness that typically characterize wind and power data [27]. This approach shows an acceptable accuracy only for near forecasting horizons (within 6 h ahead), and error increases as the estimates get further ahead in time [28,29]. Another direct framework that can overcome the drawbacks of the traditional direct approach in WPF is the wind-to-power (W2P) approach. This method uses meteorological data obtained from NWP models to predict wind power directly. The superiority of the W2P approach over time series analysis has been demonstrated, especially for forecasting horizons greater than 6 h. Nonetheless, its precision heavily relies on the quality and resolution of the NWP model used in addition to its temporal and spatial resolution [1,30,31].

Alternatively, several studies suggested using the indirect approach to improve forecast accuracy. This approach consists of two parts. Firstly, the expected wind profiles at the site at a given timeframe are forecasted and then are transformed into wind power using suitable wind-to-power performance models (power curves).

Wind power is typically related to wind speed through a cubic relationship, implying that minor changes in wind speed induce substantial variations in wind power output. Accordingly, the indirect approach's error is composed of two terms: the first term originated from inaccurate wind speed forecasting. The second is due to inaccuracies in the nonlinear relationship used in performance models to transform wind speed into wind power. Several studies focused on improving the wind speed forecast by implementing different techniques ranging from traditional statistical time series analysis to more advanced techniques like artificial neural networks [16,18,23,32]. Alternatively, the NWP wind forecasts can be improved by downscaling them to the specific site of interest [19,33,34]. Correlating the wind speed to power can be done using the wind turbine's power curve or more efficiently using statistical and intelligent techniques [[35], [36], [37], [38], [39]].

Wind direction typically has less impact on power output than wind speed since wind turbines automatically face the wind direction during operation. As a result, wind direction is typically considered a less significant factor in wind power forecasting compared to wind speed. However, the power curves of the turbines within a wind farm can become heterogenous with changes in wind direction since, with the directional changes, some upstream turbines can become downstream ones. Therefore, it is interesting to incorporate the wind direction in developing site-specific farm-level wind-to-power performance models [40,41].

The use of NWP is crucial to obtain an accurate WPF, especially for medium-to long-term horizons [13]. However, the performance of NWP models varies significantly depending upon several factors related to the physical and numerical implementations of the atmosphere's dynamics inside the models. Hence, compared to the use of a single NWP model, the implementation of several NWP models as a combination has proved to improve the performance of WPF [6,42].

WPF models are typically developed at the farm-level, which can include several input features from NWPs and lagged measurements [27,29,43,44]. In contrast, some studies propose that WPF can be made at individual turbine-level, which can then be aggregated to estimate wind farm power production [1,45]. However, studies comparing the relative merits of these two methods are scarce.

This paper comprehensively presents and compares different approaches to wind farm power forecasting. It is a continuation of a previous study [31]. The methodology developed in that study for WPF at the turbine level is here applied to develop models for WPF at the farm level. The new farm-level results are compared with the previous aggregated turbine-level results, allowing a unique and comprehensive comparison between aggregated turbine- and farm-level approaches. Short-to medium-term forecasting schemes are utilized to meet the trade requirements of ongoing intraday and day-ahead markets of the Nord Pool. Features of the current study, distinguishing it from previous research, are highlighted below:

• **Comparison between farm- and aggregated turbine-level forecasting approaches:** As previously mentioned, most of the reported studies suggest using farm-level power forecasting, whereas few studies indicated similar or even slightly better performance for the aggregated turbine-level forecasting approach. As a proper comparison between these methods is not available in the literature, we compare both these approaches considering the same wind farm, period, forecasting horizon, modelling techniques and post-processing methods.
• **Integrating inputs from several NWPs at farm-level WFP:** Weather parameters from three different NWP models with various spatial resolutions are used for the proposed WPF. The different NWP-based WPFs thus developed are then combined to form a single WPF which could exploit the strengths of different NWPs. Such an approach is rarely seen in the literature.
• **Comparison between the direct and indirect farm-level WPF:** There are disagreements in the literature on whether the direct or indirect approach would give the best results in forecasting. According to some studies [46], the direct WPF approach has been found to provide better accuracy than other methods for 1-h ahead time horizon. At the same time [12,47], favour the indirect approach for 24–48 h ahead. In this study, we will assess the accuracy of both the direct and indirect WPF methods by applying them to the same wind farm and evaluating their forecasting performance for power output at both the aggregated turbine-level and farm-level.
• **Investigating wind direction influence on farm-level WPF model performances:** Considering the effect of wind direction on the interaction between the turbines and, thereby, on the power generation, we propose specific WPF models for different directional sectors of wind. These are then compared with the traditional approaches.

The remainder of the paper is organized as follows. The data and pre-processing procedures used are shortly described initially since the raw data set is the same as the one used in Ref. [31]. Next, we will present the methods utilized to create and optimize the farm-level WPF models, which will be briefly summarized since the methodology is similar to the one used in Ref. [31]. After this, the performance of farm-level models is discussed and compared with the aggregated turbine-level results in Ref. [31]. Lastly, results are summarized, and main conclusions are highlighted.

## 2 Data description and pre-processing

**Fig. 1:**
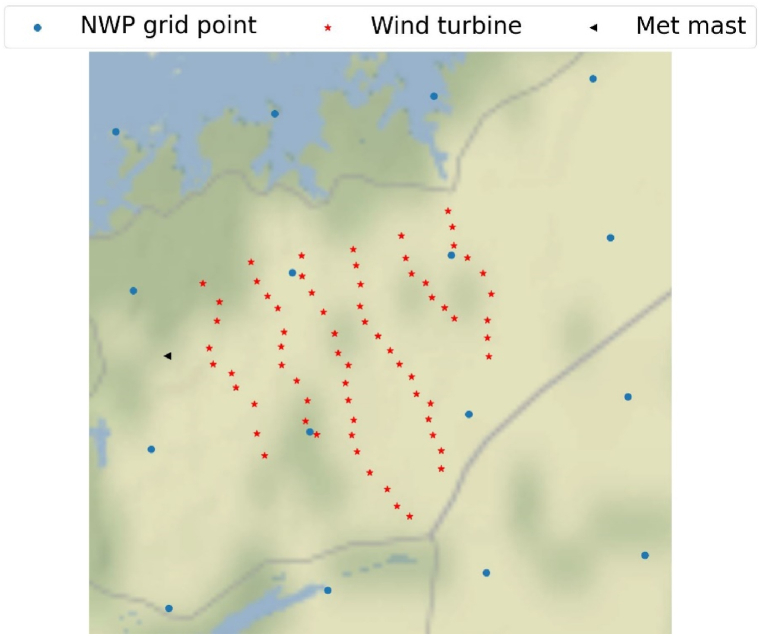
Wind turbines, met mast, and NWP grid points with 2.5 km × 2.5 km resolution.

Smøla wind farm in Norway is used as a case study. The farm comprises 68 wind turbines (see Fig. 1) with different rotor diameters of 76 m (2 MW) and 82.4 m (2.3 MW) installed over 70 m towers (for more details, see Ref. [31]). The data set is the same as used in Ref. [31], comprising production and wind speed measurements from the wind farm's SCADA system aggregated to the hourly average for the period from January 2017 to December 2020.

**Fig. 2:**
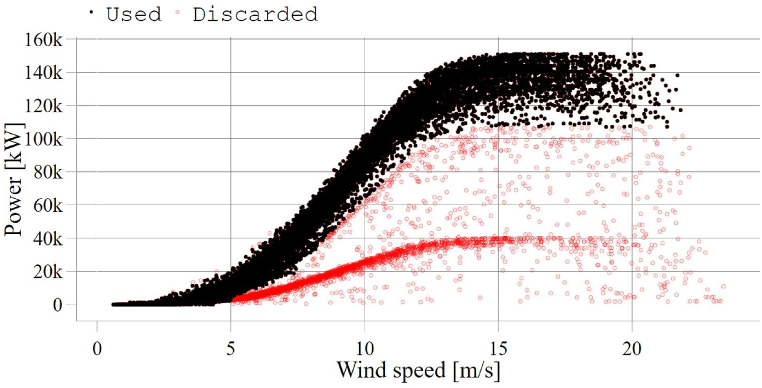
Used data versus discarded data (farm-level).

As discussed in Ref. [31], outliers caused by turbine downtime, sensor malfunction, signal noise, power curtailment and icing were identified and removed while pre-processing (cleaning) the turbine-level data. These outliers represented approximately 15 % of the record on the wind farm level, as shown in Fig. 2.

In addition, historical weather forecasts for the wind farm location from 3 NWP archives were extracted over the studied period. Table 1 summarizes the main characteristics of the NWP sources used, and Fig. 1 presents the NWP grid coordinates from the high-resolution model (MEPS) within and nearby the wind farm (for more details, see Ref. [31]).

**Table 1:** Main characteristics of the NWP sources.

NWP model Name	Temporal resolution	Spatial resolution	Update rate	Update time
**ECMWF-ifs**^a^**(IFS)** [48]	1-h	7.6 km	2/day	06 and 18 UTC
**Ukmo-euro4**^b^**(EURO)** [49]	1-h	3.05 km	4/day	00, 06, 12 and 18 UTC
**MEPS**^c^ [50]	1-h	2.5 km	4/day	00, 06, 12 and 18 UTC

a European Centre for Medium-Range Weather Forecasts.

b UK MetOffice

c MetCoOp Ensemble Prediction System.

SCADA (for each turbine) and NWP archives historical data was divided into two sets: i) calibration set, which included train and validation data, and ii) testing set. The latter is composed of the initial 10 days of each month (representing about 30 % of the complete dataset) while the remaining data was used for calibration. The inclusion of consecutive days in the test set was necessary to estimate errors over at least 36 consecutive hours, which is relevant for the ‘Elspot’ market in Norway.

**Fig. 3:**
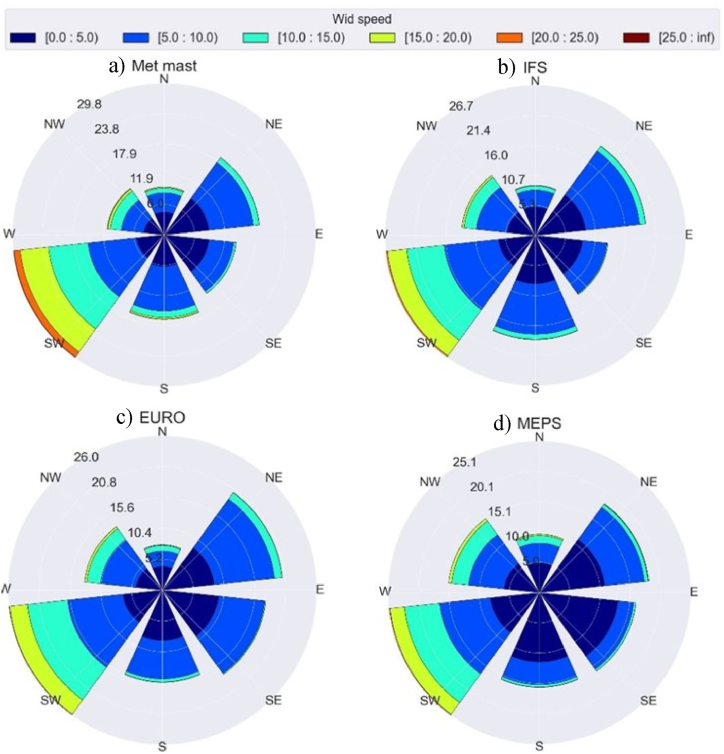
Wind rose diagrams based on observations: a) met mast, and on NWP forecasts (average over the grid points): b) IFS, c) EURO, and d) MEPS.

The wind roses based on the hourly wind data for the studied period, using both the met mast observations and the NWP forecasts at the hub height (except for the MEPS model), are shown in Fig. 3. The weather forecasts from the three NWP models resemble the observed wind conditions, indicating a good forecasting performance. According to the observations, wind has a dominant direction (West/South-West), which has a frequency of approximately 30 % of the time. Moreover, by examining the wind from the West/South-West direction, it can be read that the wind blows at speeds below 5 m/s 5 % of the time, at speeds between 5 and 10 m/s 9 % of the time, at speeds between 10 and 15 m/s 8 % of the time, and at speeds between above 15 m/s 8 % of the time.

## 3 Methods

### 3.1 Forecasting strategies

**Fig. 4:**
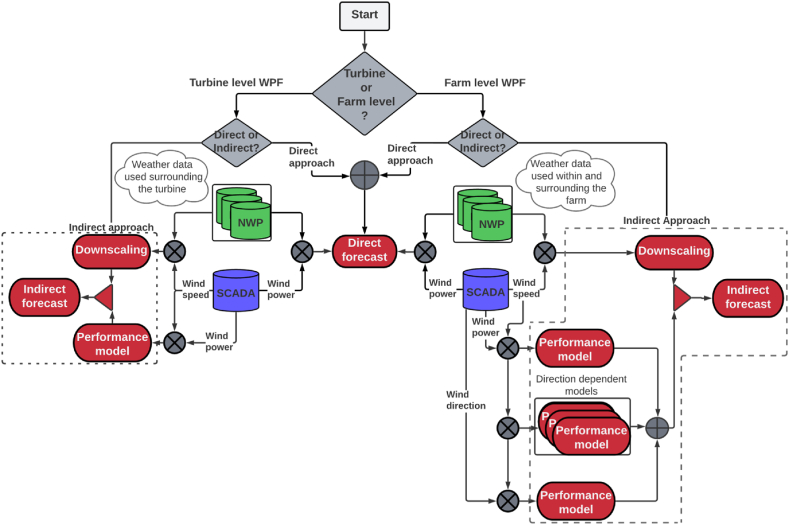
The proposed forecasting structure.

The proposed forecasting structure is schematically presented in Fig. 4. It comprises forecasting schemes considering both turbine- and farm-level while adapting both direct and indirect forecasting approaches.

In the turbine-level forecasts, weather parameters, wind speed (WS), wind gust (WG), wind direction (WD) and temperature at the nearest 4 grid cells to the turbine location were used to develop the forecasting models in both the direct and indirect approaches (this was previously done in Ref. [31]). Whereas in the farm-level forecast, the above weather parameters from all the grid cells surrounding the farm location were used (see Fig. 1).

The direct WPF scheme correlates weather parameters directly with power production measurements from SCADA. This means that the weather parameters from the relevant NWP grid cells are used to predict power output without intermediate steps.

The indirect WPF approach involves two steps: first, a correlation is established between the weather parameters and the nacelle's wind speed measurements to create a wind speed downscaling model. Then, this model is utilized to predict the wind speed at the turbine hub height. Second, the turbine's (or farm's) wind speed-power performance model is used to derive the final WPF based on the predicted wind speed.

Wind speeds at the turbine location were downscaled using NWP data from the nearest 4 grid cells for the turbine-level WPF (see details in Ref. [31]). For the farm-level WPF, the average wind speed of all wind farm turbines was downscaled to the farm location using NWP data from all the grid cells within and surrounding the farm location (see Fig. 2).

Three different approaches were considered and compared for including wind direction as an input to the model under the farm-level WPF, as shown in Fig. 4. Initially, the entire historical record of the observed wind speeds (WS_obs_) was utilized to model the wind farm power (WP_farm_). Here, the wind speed is the average of the wind turbines' nacelle measurements. In the second approach, both observed wind speed and direction (entire historical record) were used to model the wind farm power. As the yaw angle data for several turbines were missing or corrupted, wind direction measurements from the nearby existing met mast (see Fig. 1) were used as the model input. In the third approach, the data were divided into six sectors according to the wind direction. A performance model was developed for each sector using the sector's averaged observed wind speed. The number of sectors was chosen as six to ensure the diversity of conditions shown in the wind roses (Fig. 3) and the availability of sufficient data to develop the ML models for each sector.

Like in Ref. [31], the developed forecasts here use weather data from three NWP sources used individually and together (MIX), resulting in several NWP-based WPFs. Thus, for the MIX models, the number of weather inputs was three times more compared with using a single NWP source. The methodology for developing the NWP-based WPFs using either direct or indirect approaches is common for both turbine- and farm-level forecasting, so each would have 4 direct WPFs, 4 indirect WPFs, plus 3 WPFs resulting from combining the results of the direct WPFs based on the 3 NWP sources, from combining the results of the indirect WPFs based on 3 NWP sources, and from combining the results of the two previous combined WPFs (see Ref. [31] for more details). According to Ref. [31], the combined forecasts are obtained by assigning a weighting to each of the different forecasts based on their performance in the previous hours. The predicted value of a given approach (direct or indirect) using multiple NWPs is then determined.

(1) $${\hat{P}}_{t+\Delta t}=\sum {\left({W_{{App}_{j}}^{{NWP}_{i}}*\hat{P}}_{{App}_{j}}^{{NWP}_{i}}\right)}_{t+\Delta t}$$

where ${NWP}_{i}\in \left[IFS,EURO,MEPS\right]$, ${App}_{j}\in \left[Direct,Indirect\right]$ and W are the weights. For each forecasting model, the weighting multiplier was computed using the known *RMSE* for the previous 6 h of all forecasting models. Thus,

(2) $${W_{{App}_{j}}^{{NWP}_{i}}}_{\left(t+\Delta t\right)}=\frac{{\left({{RMSE}_{{App}_{j}}^{{NWP}_{i}}}_{\left(t-6\right)}\right)}^{-2}}{\sum {\left({{RMSE}_{{App}_{j}}^{{NWP}_{i}}}_{\left(t-6\right)}\right)}^{-2}}$$

So, small *RMSE* models get bigger weighting multipliers *W* (i.e., contribute more to the predicted power in Eq. (1)), and vice versa. The forecast horizon Δt is chosen to be 2 h, matching the intraday ‘Elbas' market in Norway.

Concerning the operationality of the forecasts, Table 2 shows the relative forecast horizon, market and time of generation.

**Table 2:** Forecast horizon and time resolution.

Market	forecast horizon	time resolution	time of forecast generation
Day ahead	12–36 h	1-h	12 UTC
Intraday	2 h ahead	1-h	06,12,18,00 UTC

Given the available historical raw NWP forecasts, our utilization is limited to the most recent update for each NWP forecast. For instance, data sourced from the 06:00 UTC update encompasses only the subsequent 6 h within the archive. To be precise, the models developed in this study are tailored to the intraday market. In the context of the day-ahead market, the fitting process benefits from the utilization of the most updated NWP results. Despite this, it is worth noting that NWP results generally experience minimal degradation with a lead time generation of 36 h as concluded in Ref. [45]. Consequently, while the day-ahead error is anticipated to be higher compared to the presented figures, it is still expected to fall within a similar order of magnitude.

### 3.2 Development of the forecasting model

To develop the aforementioned forecasts, we utilized a fully automated process, as described in Ref. [31], the method involves a tree-based genetic programming (GP) to find the optimal ML pipeline. This pipeline is composed of several techniques and algorithms related to feature processing, model selection, and parameter optimization. The list of regression models and data processing techniques utilized in the GP configurations can be consulted in Ref. [31]. Each of the models and techniques utilized in this process has a variety of hyperparameters that are fine-tuned during the search. A comprehensive outline of the models and techniques employed can be found in Refs. [51,52].

Different metrics were used to assess the performance of the developed forecasts (models), namely: root mean squared error (*RMSE*), normalized *RMSE* (*NRMSE*), mean absolute error (*MAE*), normalized *MAE* (*NMAE*), coefficient of determination (*R*^2^), and the index of agreement (*IoA*). The mathematical formulation for these metrics can be found, for example, in Ref. [31]. RMSE and MAE are widely used metrics for evaluating the disparities between predicted and measured values. Normalizing those metrics is helpful when comparing different case studies (i.e., wind farms). Whereas *R*^2^ assesses the goodness of fit, i.e., how well the model can forecast the observed data. In the same pattern, *IoA* is a standardized measure utilized to assess the degree of agreement between predicted values and observed values in a model's performance evaluation.

## 4 Results and discussion

### 4.1 Aggregated turbine-level WPF

As mentioned previously, the present paper is a continuation of a previous study [31], where the authors investigated the use of multiple NWP sources in wind power forecasting for individual wind turbines and for the wind farm by aggregating the individual results. In total, 8 turbine-level WPFs for each of the studied farm's turbines were developed by adapting the methodology described in Ref. [31]. For the turbine-level WPFs, the following main findings from that study are:

• Concerning the downscaling part of the indirect approach, all the developed NWP-based downscaling models, named MIX, IFS, EURO, and MEPS, enhanced the spatial resolution of the raw NWP wind predictions to the turbine-hub coordinates. On average, across all the turbines, the downscaling models showed low *RMSE* values corresponding to 1.30, 1.41, 1.51 and 1.66 m/s, respectively. The downscaling models reduce the RMSE of the NWP raw wind forecasts from IFS, EURO, and MEPS by 15 %, 11 % and 22 %, respectively.
• Each turbine's site-specific wind speed-power performance model showed low error indices, implying that the proposed ML algorithms can model the turbines' performances. On average, the performance models have *NRMSE* and *NMAE* below 2.5 % and 1.5 % across the turbines.
• Combining the forecasts from multiple turbines into an aggregate forecast renders lower overall errors compared to individual turbine forecasts. This is because errors in the predictions for each turbine can partially cancel each other out when their power outputs are combined. No conclusive evidence suggests that either the direct or indirect WPF consistently outperforms the other. The aggregated wind farm power forecasts (MIX, IFS, EURO, and MEPS) showed similar performances (see Table 2). For example, in the corresponding direct approach, *NRMSE* values were 7.8 %, 8.7 %, 9.2 % and 10 %, respectively, with negligible differences compared to the indirect approach.
• The combined forecast in both the direct and indirect approaches demonstrated performance improvements compared to the single NWP-based WPFs (IFS, EURO, and MEPS). The results are similar to the MIX forecast. Furthermore, both the combined forecasts showed superior performance compared to all other alternatives with a single NWP-based WPF, as shown in Table 3.

**Table 3:** Performance evaluation of aggregated turbine-level WPFs from Ref. [31].

Wind farm power forecasts based on aggregated turbine-level forecasts											
Error metric [MW]	MIX		IFS		EURO		MEPS		Combined		
	Dir	Indir	Dir	Indir	Dir	Indir	Dir	Indir	Dir	Indir	Both
***RMSE***	11.76	11.90	13.12	13.24	13.89	14.12	15.04	15.11	12.08	12.05	11.98
***NRMSE* [%]**	7.82	7.91	8.73	8.80	9.23	9.39	10.0	10.05	8.03	8.01	7.97
***MAE***	6.99	6.81	7.84	7.58	8.23	8.02	9.48	8.82	7.34	6.98	7.06
***NMAE* [%]**	4.65	4.53	5.21	5.04	5.48	5.33	6.31	5.86	4.88	4.64	4.69
***IoA* [%]**	97.5	97.5	96.9	96.9	96.5	96.5	95.6	95.8	97.3	97.4	97.4
***R***^**2**^**[%]**	91.0	90.8	88.8	88.6	87.4	87.0	85.3	85.1	90.5	90.6	90.1

### 4.2 Farm-level WPF

The subsequent subsections will discuss the performance of the proposed WPF models at the farm level for both the direct and indirect forecasting approaches. Eight models were developed and combined depending on the approach and the NWP weather data used. In addition to that, an analysis of implementing the wind direction in modelling the wind farm performance will be presented as part of the indirect approach.

#### 4.2.1 Farm-level wind downscaling models for indirect WPF

This subsection presents and discusses the results of the farm-level wind-downscaling in the indirect forecast scheme, which includes a wind downscaling model and a farm's site-specific performance model. Table 4 shows the performance of the farm-level wind downscaling models, which improves the wind predictions from the NWPs. When compared to downscaling models based on a single NWP, the accuracy of downscaling is enhanced using all three NWPs together (referred to as MIX), resulting in reduced error. The degree of improvement varies, with a range of 8 %, 14 % and 22 % compared to the downscaling models based on IFS, EURO, and MEPS, respectively. This is identical to the improvements achieved by adapting the turbine-level downscaling models' scheme [31]. However, at the farm-level, the wind speed downscaling models demonstrated a reduction in error of approximately 7.5 % due to the cancellation of errors. This occurred because predictions from all NWP grid cells within and nearby the wind farm were utilized.

**Table 4:** Farm-level wind speed downscaling and NWPs wind speed evaluation results.

Metric	Wind Speed Downscaling				Raw Wind Speed Forecast		
	MIX	IFS	EURO	MEPS	IFS	EURO	MEPS
***RMSE* [m/s]**	1.20	1.31	1.39	1.54	1.44	1.46	1.92
NRMSE* [%]	0.07	0.08	0.09	0.1	0.09	0.09	0.12
***MAE* [m/s]**	0.91	0.99	1.05	1.19	1.1	1.11	1.52
NMAE* [%]	0.06	0.06	0.07	0.07	0.07	0.07	0.09
***IoA* [%]**	0.97	0.96	0.96	0.94	0.96	0.96	0.92
***R***^**2**^**[%]**	0.89	0.87	0.85	0.82	0.84	0.84	0.72

*: Values normalized by the rated wind speed (16 m/s).

In comparison with the raw NWPs, the results show that the downscaling models lead to an improvement in the raw wind forecasts from IFS, EURO, and MEPS by approximately 9 %, 5 %, and 19 %, respectively. Notably, the MEPS model demonstrates the highest level of performance improvement, indicating that the downscaling technique enhances wind forecasting accuracy in both the horizontal plane and vertical coordinates, since MEPS data refers to 10 m above the ground, while all the others refer to 70 m above the ground (i.e. turbines' hub). It should be mentioned that in this comparison, an average raw NWP wind speed from all the grid cells and an average of observed nacelle's wind speeds were used.

#### 4.2.2 Farm-level site-specific performance models for the indirect WPF

**Fig. 5:**
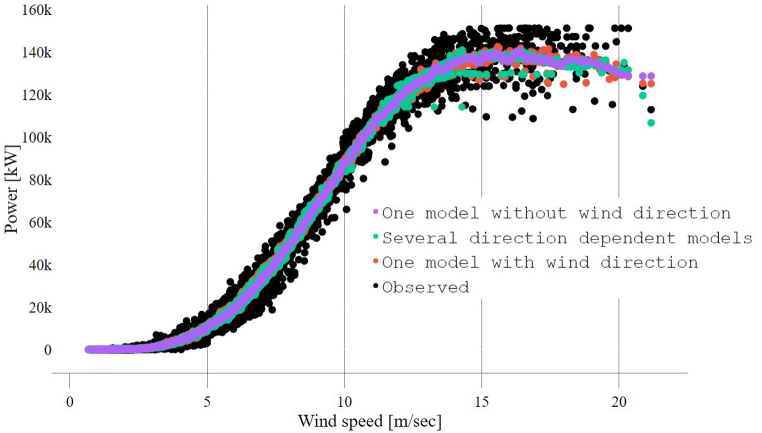
Wind farm site-specific performance models versus observations.

The performance of the site-specific wind farm models adapting the three mentioned approaches (section 3.1) can be seen in Table 5. In general, the use of wind direction, either as independent input or as an indicator of the wind sector (direction-dependent), improved the capability to model the farm's performances compared with the traditional approach (only wind speed), reducing the *NRMSE* and *NMAE* by 3.6 % and 5.9 %, respectively. The directional band-based approach's performance showed negligible improvement compared to the performance of using wind direction as an input. However, from the WPF perspective, using wind direction as input would require a downscaling model, which in turn would hypothetically add an additional error term to the overall error [19,39]. In addition, the low error index values and high *IoA* and *R*^*2*^ values confirm the well-known model's ability to predict the farm's performance. This can also be seen in Fig. 5, where the power production of the different approaches is compared with observed values. Obviously, several directional-dependent performance models introduced more variability than a single model approach.

**Table 5:** Wind farm's performance models adopting different approaches.

Inputs	Metric		
	Traditional One model	Traditional with wind direction One model	Directional dependent Several models
	WS	WS & WD	WS
**RMSE [MW]**	4.15	4.08	4.07
NRMSE* [%]	2.8	2.7	2.7
**MAE [MW]**	2.54	2.46	2.44
NMAE* [%]	1.7	1.6	1.6
**IoA [%]**	99.7	99.8	99.8
**R**^**2**^**[%]**	99	99	99

*: Values normalized by the rated wind farm power (150.4 MW).

**Fig. 6:**
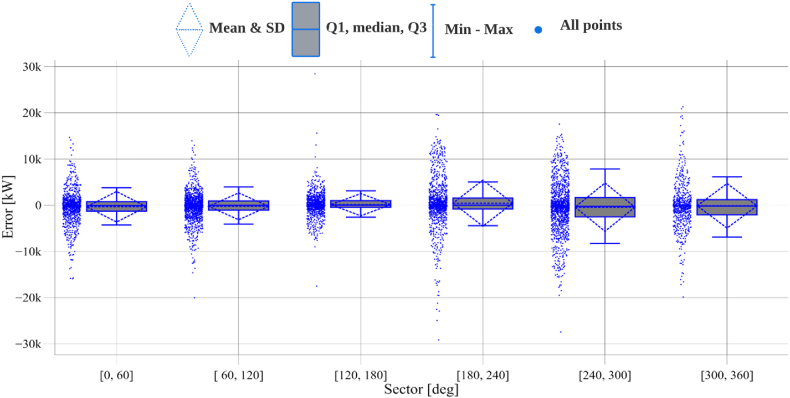
Directional dependent models error quantification.

To illustrate further, Table 6 presents the performance evaluation of the directional dependent models, where the dominant directions (most frequent) show a higher error than other sectors due to the high variations in the wind speeds, as presented in Fig. 3. The error rate can be varied by (37 %→51 %) and (41 %→58 %) considering *NRMSE* and *NMAE*, respectively, while comparing different sectors. However, an error quantification of the directional dependent models' variability can be seen in Fig. 6. All sectors have an approximate average of 0, and most error values are concentrated within the lower and upper fences. However, the sectors between 180 and 360 showed an increased number of outliers that lay outside the fence's range. This explains the increase in RMSE for these sectors, as the errors are squared before being averaged, which gives higher weight to significant errors.

**Table 6:** Performance evaluation of directional dependent models.

Metric	Wind direction sectors					
	0^°^→60^°^	60^°^→120^°^	120^°^→180^°^	180^°^→240^°^	240^°^→300^°^	300^°^→360^°^
***RMSE* [MW]**	3.326	2.923	2.411	5.009	5.247	4.825
***NRMSE* [%]**	2.2	1.9	1.6	3.3	3.5	3.2
***MAE* [MW]**	2.050	1.818	1.412	2.983	3.556	3.080
***NMAE* [%]**	1.4	1.2	0.94	2.0	2.4	2.05
***IoA* [%]**	99.7	99.7	99.7	99.7	99.7	99.6
***R***^**2**^**[%]**	98.8	99.0	98.7	99.0	98.9	98.6

It should be noted that the upper and lower fences in Fig. 6 represent the cut-off values for upper and lower outliers in a dataset (lower and upper fences).

#### 4.2.3 Farm-level wind power forecasts (indirect vs. direct)

The performances of direct and indirect farm-level forecasting models are shown in Table 7. The results are aligned with the findings of the turbine-level WPF developed previously in Ref. [31] (see Table 3). It is evident that using multiple NWP sources together (MIX) significantly improves the performance of the models. For example, the MIX could enhance the *RMSE* and *MAE* of direct forecasting models, compared to single NWP-based WPFs, by 10 %–24 % and 11 %–30 %, respectively. Similar improvements, 8 %–22 % and 8 %–23 %, were obtained for indirect forecasts. In the case of IoA and R^2^, similar improvements in performance can be observed, although they are of a smaller magnitude.

**Table 7:** Performance evaluation of farm-level WPFs.

Farm-level Wind Power Forecasts											
Metric	MIX		IFS		EURO		MEPS		Combined		
	Dir	Indir	Dir	Indir	Dir	Indir	Dir	Indir	Dir	Indir	Both
***RMSE* [MW]**	13.29	13.48	14.82	14.68	15.70	15.60	17.51	17.44	14.10	14.00	13.87
***NRMSE* [%]**	8.83	8.96	9.85	9.76	10.44	10.38	11.64	11.60	9.38	9.31	9.22
***MAE* [MW]**	8.37	8.23	9.48	8.97	9.62	9.48	11.84	10.70	8.76	8.36	8.39
***NMAE* [%]**	5.56	5.47	6.30	5.96	6.40	6.30	7.88	7.11	5.82	5.56	5.58
***IoA* [%]**	97.03	97.03	96.27	96.47	95.91	95.99	94.33	94.78	96.57	96.72	96.74
***R***^**2**^**[%]**	89.38	89.06	86.77	87.01	85.15	85.33	81.53	81.68	88.02	88.19	88.41

As found in Ref. [31] for the turbine-level, also on the farm-level the WPF model that utilizes the lowest spatial resolution NWP (IFS) showed better performance in comparison to the ones using higher spatial resolution NWPs (namely, EURO and MEPS), the enhancements obtained in terms of RMSE and MAE range from 5 % to 16 % in both the direct and indirect approaches.

**Fig. 7:**
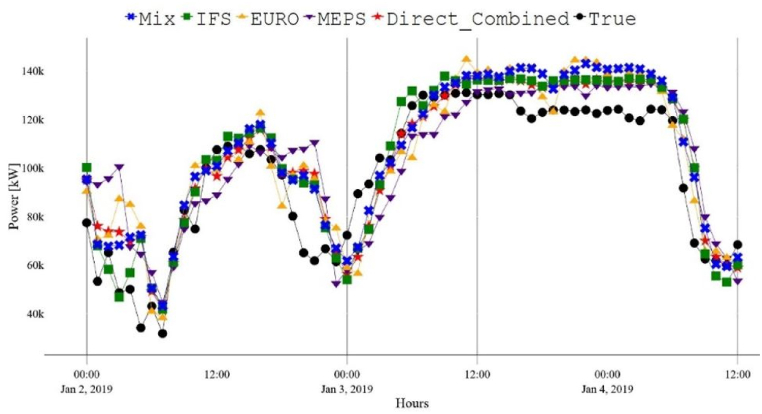
Direct WPFs between the 2nd and January 4, 2019.

**Fig. 8:**
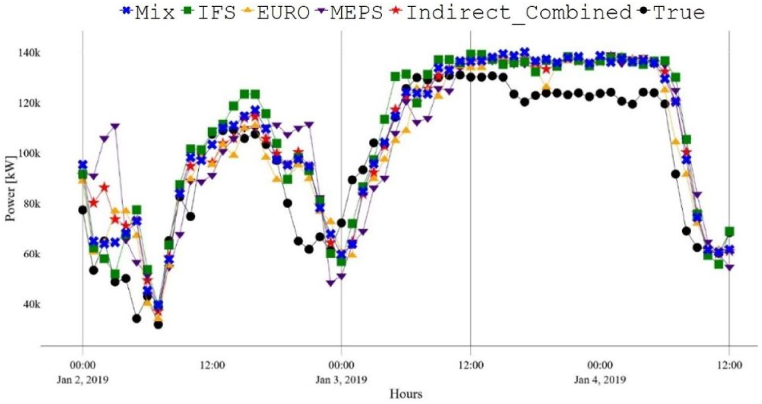
Indirect WPFs between the 2nd and January 4, 2019.

Furthermore, the combined forecast, obtained by combining the three developed WPFs using equations (1), (2)), demonstrated enhanced performance in each approach and when considering both approaches together (resulting in a total of six WPFs). The performance improvement was comparable to that of the MIX forecast. However, the MIX forecast slightly outperformed the others with improved RMSE and MAE values below 5 %. These results confirm that utilizing multiple NWPs leads to superior overall forecasts. Furthermore, both the direct and indirect wind farm-level power forecasts demonstrated comparable performance for the wind farm analyzed in this study, which differs from what has been reported in some previous studies. As an illustration, while [46] suggests that the direct approach is more accurate than the indirect one [47], argues the opposite. Nonetheless, the performance variations for all models between the two approaches are minimal, with differences of less than 2 % across various error metrics. This is clearly depicted by comparing Fig. 7, Fig. 8.

**Fig. 9:**
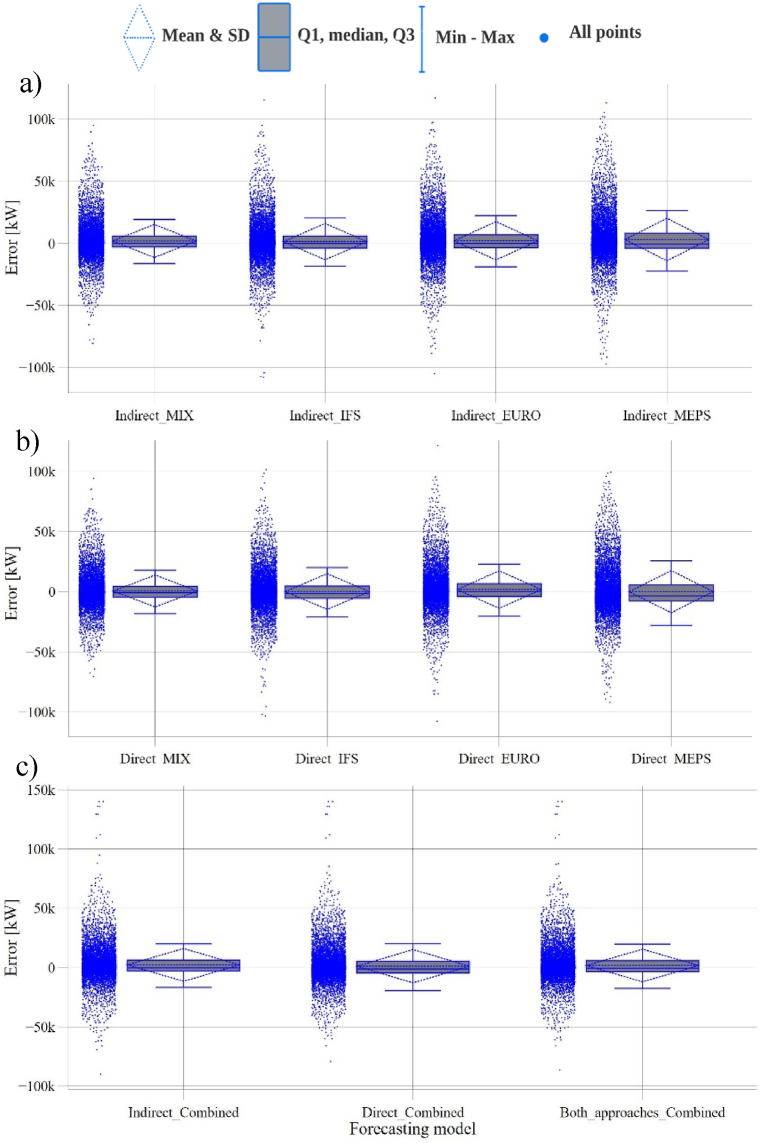
Wind farm WPFs' error quantification: a) indirect models, b) direct models, and c) combined models.

**Fig. 10:**
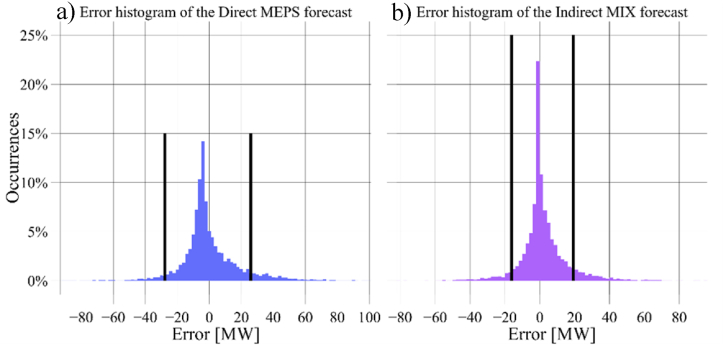
Error histogram for two of the developed forecasts: a) Direct MEPS, and b) Indirect MIX.

Table 8 and Fig. 9 provide a detailed analysis of the error characteristics of the developed wind farm-level forecasts. The errors, defined as the difference between observed and predicted values, exhibit negative medians and positive means. The former indicates that more than half of the predictions are slightly overestimated, while the latter implies that the remaining predictions are underestimated but with larger magnitudes. Additionally, the forecasts demonstrate a right-skewed distribution, being the medians closer to the first quartile (Q1) than to the third one (Q3), except for the direct-MIX forecast, which exhibits a more symmetrical distribution. The MEPS forecasts show the widest interquartile ranges (IQR), exceeding 12 MW. This indicates that the predictions from the MEPS model are more dispersed and have a broader range of variability compared to other forecasts, as supported by the comparison of standard deviations (SD). However, it is worth noting that approximately 90 % of the errors fall within the fence ranges for all the WPFs, as depicted in Fig. 9. Furthermore, Fig. 10 illustrates the error occurrence histograms for two forecasts, considering the smallest and largest fence ranges.

**Table 8:** Error quantification of the developed farm-level WPFs.

Wind farm-level error quantification											
Error [MW]	MIX		IFS		EURO		MEPS		Combined		
	Dir	Indir	Dir	Indir	Dir	Indir	Dir	Indir	Dir	Indir	Both
**Mean**	0.65	1.99	0.21	1.46	1.86	2.16	0.11	3.15	1.21	2.32	1.80
**Median**	−0.77	−0.04	−1.34	−0.09	−0.16	−0.06	−3.39	−0.45	−1.05	−0.19	−0.55
**SD**	13.27	13.33	14.82	14.61	15.59	15.45	17.50	17.15	14.05	13.81	13.75
**Q1**	−4.59	−2.88	−5.47	−3.79	−3.98	−3.35	−7.70	−3.91	−4.52	−2.87	−3.53
**Q3**	4.46	5.98	4.82	5.98	6.83	6.97	5.75	8.29	5.39	6.35	5.86
**IQR**	9.05	8.90	10.29	9.77	10.81	10.32	13.45	12.2	9.91	9.22	9.39
**Upper fence**	18.02	19.21	20.19	20.57	23.02	22.45	25.91	26.59	20.25	20.17	19.90
**Lower fence**	−18.13	−16.18	−20.88	−18.42	−20.17	−18.83	−27.85	−22.19	−19.39	−16.64	−17.59

### 4.3 Farm-level versus aggregated turbine-level WPF

This sub-section compares the performance of WPFs based on turbine- and farm-level. Table 3, Table 7 quantify the developed models' performance in estimating the farm's power generated based on aggregating the turbine-level power estimates and the farm-level, respectively. The aggregated turbine-level WPFs exhibited lower overall errors compared to those obtained for farm-level WPFs, and clear improvements can be observed in Table 9. In general, the improvements observed were over 10 % and 15 % for *RMSE* and *MAE*, respectively, across the different forecasts. However, the MEPS models showed the most significant improvements compared to other models.

**Table 9:** WPFs performance improvements by using the aggregated turbine-level compared to farm-level.

Performance Improvements											
Δ_Metric_ [%]	MIX		IFS		EURO		MEPS		Combined		
	Dir	Indir	Dir	Indir	Dir	Indir	Dir	Indir	Dir	Indir	Both
**Δ**_***RMSE***_	11.5	11.7	11.5	9.8	11.5	9.5	14.1	13.4	14.3	13.9	13.6
**Δ**_***MAE***_	16.5	17.3	17.3	15.5	14.4	15.4	19.9	17.6	16.2	16.5	15.9
**Δ**_***IoA***_	0.5	0.5	0.7	0.4	0.6	0.5	1.3	1.1	0.8	0.7	0.7
**Δ**_***R*2**_	1.8	2	2.3	1.8	2.6	2	4.6	4.2	2.8	2.7	1.9

**Fig. 11:**
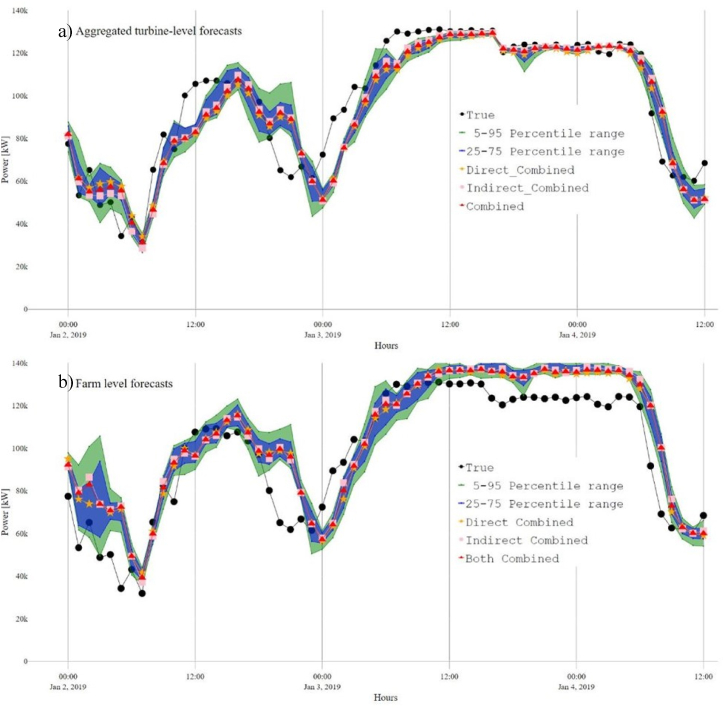
Wind power forecasts with prediction intervals: a) aggregated turbine-level, and b) versus farm-level.

Moreover, the indirect approach models were less improved than the direct ones except for the MIX models, where the results were similar, reflecting the improvement achieved by the wind speed downscaling models on the wind farm level. This is further shown in Fig. 11, where the percentile ranges from the predictions based on the different models (MIX, IFS, EURO, and MEPS) are also illustrated. Aggregating the forecasts resulted in lower overall errors compared to wind farm-level forecasts, as the over- and under-predictions of individual turbine power outputs could partially offset each other. Similar improvements in accuracy measures, such as *R*^2^ and *IoA*, can also be observed. Furthermore, both the IQR and the fence range for the farm-level WPFs are broader than those for the aggregated farm forecasts based on the turbine-level [31], proving that aggregated turbine-level forecasts have less dispersed and a lower error magnitude than farm-level forecasts.

## 5 Conclusions

This study examines the importance of utilizing multiple NWP sources in wind power forecasting (WPF) for wind farm-level forecasts while considering both direct and indirect forecasting approaches. These forecasts are further compared with aggregated turbine-level forecasts (developed previously by the authors in Ref. [31]) to investigate the advantages of each framework. Different approaches were implemented in modelling the site-specific wind farm performance model, considering wind direction either as individual input or as an indicator of the direction of sector-based models. The construction of the prediction models involved employing a tree-based genetic programming optimization search to identify the optimal machine-learning pipeline. The conclusions of the study can be summarized as follows:

• The downscaling models at both farm- and turbine-level improved the raw NWP. However, the farm-level downscaling model had a smaller error magnitude due to error cancellation as several NWP grid cells were utilized.
• The use of wind direction in modelling the wind farm performance, either as independent input or as a sector indicator, showed similar performance, which in turn would result in negligible improvements in the indirect WPF.
• Utilizing multiple NWP models produces more accurate forecasts than relying on a single NWP, regardless of whether being integrated into one model as in the MIX or into a combination of several WPF NWP-based models as in the combined WPFs.
• The accuracy of the farm-level WPF, whether using the direct or indirect approaches, was improved by using multiple NWPs.
• There is no conclusive evidence supporting the superiority of either the direct or indirect WPF approach over the other. Therefore, a more complex terrain may be used to assess the performance of direct and indirect WPFs.
• For the direct approach, the use of aggregated turbine-level WPF improved the accuracy across the different forecasts compared with farm-level WPFs. However, that improvement is less pronounced for the indirect approach due to the improved downscaling models at the farm-level.
• The error analysis showed that 50 % of the predictions are within the IQR, while 90 % are within the fence ranges. However, these ranges are wider than the ones for the aggregated farm forecasts based on turbine-level WPFs.

Resuming direct turbine-level WPFs using several NWPs (MIX), aggregated for the whole farm, resulted in the best forecasting accuracies among the compared WPF schemes. The use of several NWPs significantly contributed to enhancing forecasting accuracies. Though the indirect models could also show reasonable performances, the need to build two models (wind downscaling model and the turbine performance model) for each turbine can make this approach more complex. Although the aggregated turbine-level approach requires much more development work (at least one model per turbine) than the farm-level approach, it is justified by the improvements in model performance. Further, aggregated turbine-level forecasting schemes are not affected by operational issues like the shutdown of individual turbines or restrictions due to power curtailments, which is an added advantage of this method. The ongoing extension of the study to wind farms with more complex terrains aims to establish the applicability of the proposed methods across diverse wind farm topographies. Furthermore, several potential avenues for future research have been identified, such as enhancing the integration of multiple NWP models within a probabilistic framework, exploring the benefits of optimizing the selection of NWP grid points and weather variables, evaluating the economic feasibility of these forecasts, and applying the developed method to larger onshore and offshore wind farms.

## Data availability statement

Data disclosure is binded by a non-disclosure agreement.

## CRediT authorship contribution statement

**Ghali Yakoub:** Conceptualization, Formal analysis, Methodology, Software, Validation, Visualization, Writing – original draft. **Sathyajith Mathew:** Supervision, Writing – review & editing. **Joao Leal:** Supervision, Writing – review & editing.

## Declaration of competing interest

The authors declare that they have no known competing financial interests or personal relationships that could have appeared to influence the work reported in this paper.
